# Metabolome Combined with 16S rDNA Sequencing Reveals a Novel Mechanistic Insight into the Collaboration of Resveratrol and β-Hydroxy-β-Methylbutyric Acid in Regulating the Meat Quality of Tibetan Sheep Through Altering Rumen Microbiota

**DOI:** 10.3390/microorganisms13122845

**Published:** 2025-12-15

**Authors:** Jiacheng Gan, Qiurong Ji, Kaina Zhu, Zhenling Wu, Xuan Chen, Shengzhen Hou, Linsheng Gui, Chao Yang

**Affiliations:** 1 State Key Laboratory of Plateau Ecology and Agriculture, Qinghai University, Xining 810016, China; 15003623399@163.com (J.G.); qhdxhsz@163.com (S.H.); 2 College of Agriculture and Animal Husbandry, Qinghai University, Xining 810016, China; 15291938937@163.com (K.Z.); w15165514762@163.com (Z.W.); 17318583359@163.com (X.C.); gls514188@126.com (L.G.); 3 Comprehensive Service Center for Agriculture, Animal Husbandry, Science and Technology, and Rural Revitalization of Madoi County, Golog Tibetan Autonomous Prefecture 813500, China; jqr1960742393@163.com

**Keywords:** meat quality, metabolites, rumen microbiota, Tibetan sheep

## Abstract

Resveratrol (RES), a natural polyphenol, and β-hydroxy-β-methylbutyric acid (HMB), a key metabolite of leucine, are emerging as potent feed additives in animal production. This study investigated the individual and combined effects of dietary RES and HMB on gut microbiota, metabolic profiles, and meat quality in Tibetan sheep. A total of 120 two-month-old male lambs were randomly assigned to four experimental groups: control (C, basal diet, n = 6), RES (basal diet + 1.5 g/d RES, n = 6), HMB (basal diet + 1.25 g/d HMB, n = 6), and RES-HMB (basal diet + 1.5 g/d RES + 1.25 g/d HMB, n = 6), with 16S rDNA sequencing and LC-MS/MS analyses performed on rumen fluid and longissimus lumborum (LL). Meat quality improved significantly in all supplemented groups, the RES-HMB co-administration exhibited the most pronounced effects, suggesting a synergistic interaction. These improvements were linked to the activation of amino acid (AA) and unsaturated fatty acid biosynthesis pathways, leading to increased levels of AAs and polyunsaturated fatty acids (PUFAs). Concurrently, dietary RES and HMB supplementation enriched the relative abundance of beneficial gut microbiota, notably *Christensenellaceae_R-7_group* and *Solibacillus*, which further promoted the production of short-chain fatty acids, AAs, and PUFAs. The study highlights the role of rumen microbiota in regulating muscle metabolism and meat quality, offering a new scientific basis of strategies for using green feed additives in Tibetan sheep.

## 1. Introduction

Tibetan sheep, a pivotal genetic resource adapted to high-altitude, cold environments, are fundamental to the meat and fur production systems of nomadic pastoral communities [1]. It has been demonstrated that Tibetan sheep exhibit higher ruminal concentrations of short-chain fatty acids (SCFAs)—primary end-products of microbial carbohydrate fermentation—compared to lowland breeds [2]. Furthermore, research suggests that Tibetan sheep exhibit advantages in carcass traits and meat excellence over Small-tailed Han sheep, especially when their diets are low in protein but high in energy; demonstrating their remarkable adaptability to harsh nutritional environments [3]. Accumulating evidence has demonstrated a strong association between rumen bacterial populations and meat metabolites in ruminants [4,5,6]. Adequate nutrient intake is therefore crucial not only for maintaining rumen homeostasis and function but also for optimizing the nutritional value and quality of meat [7,8]. However, traditional hay feeding during the winter and spring can significantly reduce the weight of ruminants [1], and in extreme cases, cause death [9]. In this context, strategic supplementation with concentrate feeds emerges as a practical approach to mitigate nutritional constraints and enhance production efficiency in Tibetan sheep grazing systems. However, the underlying mechanisms through which such nutritional interventions modulate rumen microbiota and subsequently influence meat quality metabolites remain inadequately explored. This knowledge gap necessitates systematic investigation to establish the causal relationships between dietary regimes, microbial ecosystems, and meat quality attributes in Tibetan sheep.

As a representative functional component in concentrate feeds, resveratrol (3,5,4′-trans-trihydroxystilbene, RES), which belongs to the stilbenoid group of compounds, occurs in plants as a polyphenolic defense compound, notably in white squash, Polygonum cuspidatum, grapes, and peanuts [10,11]. This compound is recognized for its pleiotropic biological activities, including potent antioxidant, anti-inflammatory, anti-glycemic, and anti-carcinogenic properties [12]. Research conducted on Dorper × Short-tailed Han crossbred ewes demonstrated that adding RES to their diet increasing the molar proportion of propionate and enriching the relative abundance of key cellulolytic bacteria, namely *Fibrobacter succinogenes*, *Ruminococcus albus*, and *Butyrivibrio fibrisolvens* [13]. Furthermore, RES has been shown to mitigate enteric methane production, an effect attributed to the suppression of methanogenic archaea, particularly the *Methanobrevibacter* [14]. Interestingly, Shen et al. found that dietary supplementation with 150 mg/kg RES improves meat yield percentage, meat quality, and ruminal microbiota in fattening goats [15]. Another study reported that 300–450 mg/kg RES supplementation improves Pekin duck meat via enhanced intramuscular fat, AA deposition, and modified muscle fiber profiles [16]. Collectively, these studies underscore the potential of RES as a functional feed additive for concurrently improving rumen fermentation efficiency, animal performance, and final meat quality attributes.

Similarly, β-hydroxy-β-methylbutyric acid (HMB) represents another promising concentrate feed supplement with demonstrated efficacy in ruminant nutrition. As an active metabolite of the essential branched-chain amino acid leucine [17]. Multiple beneficial effects of HMB have been reported, including enhancing sarcolemma integrity, inhibiting protein degradation, preventing cell apoptosis, increasing protein synthesis, and enhancing the expansion and specialization of muscle stem cells [18,19,20]. In ruminants, HMB is more efficiently absorbed through the rumen epithelium and gastric mucosal epithelium than through the stomach [21]. Supplementation with HMB has been shown to influence ruminal bacterial and protozoal populations in cows [22]. Specifically, HMB serves as a source of methionine (Met) for rumen microorganisms, significantly increasing acetate concentrations and protozoal populations [23]. Additionally, dietary HMB supplementation has been demonstrated to improve carcass quality, as evidenced by higher marbling scores in steers [24]. Notably, Wenshi broiler chickens fed HMB were found to exhibit higher average daily gain, increased a* (redness) levels and reduced L* (lightness) measurements in breast meat, and lower drip loss in breast muscle tissue [25]. Considering that endogenous synthesis of HMB is limited, exogenous supplementation is considered a prerequisite for eliciting its significant physiological and production-enhancing effects [26].

Previous studies have shown that dietary supplementation with RES and/or HMB can serve as feed additives for Tibetan sheep, improving their jejunal [27] and hepatic function [28], maintaining duodenal health [29], and regulating lipid metabolism [30]. However, the mechanisms through which RES and HMB influence meat quality, either individually or jointly with each other remain largely unclear, particularly in the context of Tibetan sheep. This study proposes the core hypothesis: dietary supplementation with RES and/or HMB can improve key meat quality traits of Tibetan sheep by regulating energy metabolism, and antioxidant capacity in skeletal muscle. To investigate this, the present study employed an integrated approach combining 16S rDNA sequencing and metabolomics to elucidate the impact of these additives on the rumen microbiota and the consequent metabolic shifts within muscle tissue that determine meat quality. The findings are expected to provide a scientific basis for the strategic application of these additives in Tibetan sheep nutrition, thereby contributing to the advancement of the regional livestock economy in Qinghai Province.

## 2. Materials and Methods

### 2.1. Ethics Statement

This research was carried out in alignment with the principles of the Institutional Animal Care and Use Committee of Qinghai University (QUA-2020-0709). Critters in this investigation were resided at Xiangkameiduo Ranch, Gonghe county, Qinghai province.

### 2.2. Management of Tibetan Sheep and Sample Collection

A total of 120 healthy 2-month-old Tibetan rams (mean initial weight of 15.5 ± 0.14 kg) were randomly assigned to 4 pens (30 sheep per pen) following a completely randomized design; prior to allocation, all rams were weighed individually and stratified random sampling was used to homogenize initial body weights among pens, with no significant difference in initial weights between pens (*p* > 0.05, one-way ANOVA), ensuring no systematic bias in animal distribution across the four experimental groups. The control group (C group) was fed a standard diet, while the other three groups received the basal diet supplemented with 1.5 g/d RES alone (RES group), 1.25 g/d HMB alone (HMB group), or a combination of 1.5 g/d RES and 1.25 g/d HMB (RES-HMB group), respectively. Table 1 detailed the dietary ingredients and their nutritional profile. The test reagent RES (purity > 99%) was obtained from Xian Plant Technology Co., Ltd. (Xian, China), and HMB (model 018, purity > 99%) was sourced from Jiyuan Group Co., Ltd. (Jiyuan, China).

On a dry matter basis, the concentrate-to-forage ratio in the basal diet was 7:3. Each treatment group included five replicate pens, with six Tibetan sheep each. The rams received their feed twice daily, at 8:00 A.M. and 5:00 P.M. According to the additive dose, both RES and HMB were weighed and mixed evenly in the basal diet evenly before feeding. The study consisted of a 10-day acclimatization period followed by a 90-day experimental phase. During the experimental period, all Tibetan sheep were reared under consistent environmental conditions with ad libitum access to feed and water. Feed intake was recorded daily, and the health status of the sheep was monitored to ensure their adaptation to the rearing environment and diet composition. Meanwhile, the pens were cleaned and disinfected daily, and adequate ventilation in the housing was maintained to minimize environmental stress.

After the feeding trial, 6 sheep were randomly selected from each group for slaughter, with their growth performance consistent with the average level of the respective group. Prior to slaughter, the selected sheep were fasted for 24 h and deprived of water for 2 h. After weighing, they were slaughtered by exsanguination via the carotid artery, and all procedures were conducted in strict compliance with the principles of the Institutional Animal Care and Use Committee of Qinghai University (Approval No.: QUA-2020-0709). Prior to collection, the rumen fluid was strained through four layers of cheesecloth. Simultaneously, the longissimus lumborum (LL) was removed before the muscle was converted into meat. After exsanguination, the carcasses were processed immediately. Prior to the onset of rigor mortis, the LL muscles on both sides of the carcass (between the 9th and 11th ribs) were dissected and separated. The samples were then transferred into sterile containers and stored at −80 °C in preparation for omics analysis.

### 2.3. Analysis of Meat Characteristics and Nutritional Quality

Meat quality was assessed using standard procedures 24 h after slaughter. A pH meter, calibrated using pH 4–6.86 standards, was inserted 2 cm deep into the meat and the pH reading was recorded. Meanwhile, The Minolta-ADCI instrument was used to quantify colorimetric data: b* (yellowness), a*, and L* in tested meats.

Following prior methodology [31], LL specimens were kept at −80 °C underwent dual weighing (pre- and post- 4 °C thawing over 12 h) to quantify thaw-induced reduction. Samples were then immersed in an 85 °C water bath for 20 min, cooking loss was subsequently quantified (%). All samples were placed in individual steaming bags during these processes. The MAEC-18 hydraulic unit (Nanjing Ming’ao Instrument Equipment Co., Ltd., Nanjing, China) was used to quantify water depletion from meat cubes (1 cm^3^). Meat samples, measuring 1 cm × 1 cm × 2 cm, were sliced in alignment with the orientation of the muscle fibers to determine the shear force (SF) using a Warner-Bratzler apparatus (TEX-01, Jinan X’ao Electromechanical Co., Ltd., Shandong, China). In addition, a texture analyzer (Universal TA, Shanghai, China) was utilized to evaluate various textural characteristics, including hardness, elasticity, stickiness, adhesiveness, cohesion, and chewiness.

The LL muscle nutrient content was established using AOAC standard protocols [32]. Ash content was determined by incineration of samples in a silica crucible using a muffle furnace (ThermoFisher, Waltham, MA, USA) at 550 °C for 4 h. The moisture contents were determined using the direct drying method, while the fat contents were measured via Soxhlet extraction [33], and the protein levels were assessed using the Kjeldahl method [34].

### 2.4. Real-Time Fluorescence Quantitative PCR Analysis

The LL tissues were homogenized, and RNA was isolated with the TransZol Up kit (TRAN, Beijing, China). Following RNA extraction, cDNA was synthesized using the All-in-One RT SuperMix for qPCR kit (with dsDNase) (Azaood, Beijing, China). Subsequently, cDNA was amplified via real-time PCR (Bio-Rad, Hercules, CA, USA) utilizing the Universal SYBR Green qPCR Mix kit (Azaood, Beijing, China). A two-step qPCR was run carefully following the recommended thermal cycling curve. GAPDH was employed as the internal reference gene for normalization. The relative expression levels of target genes were calculated using the 2^−ΔΔCt^ method. Primer details were present in Table S1.

### 2.5. Determination of AA and Medium- to Long-Chain Fatty Acid Profiles in Meat

For the determination of AA composition in meat, LL samples (60 mg) were homogenized with water, a methanol-acetonitrile solution (A955-4, Fisher Chemical, Waltham, MA, USA), and an internal standard solution (Sigma-Aldrich, St. Louis, MO, USA). The internal standard was a mixture of isotopically labeled amino acids at a working concentration of 50 μmol/L in methanol–water (1:1, *v*/*v*). The mixture was then vortexed for 30 s and subjected to ultrasonication in an ice bath for 10 min to extract free AAs. After centrifugation at 12,000× *g* for 15 min at 4 °C, the supernatant was collected and filtered through a 0.22 μm membrane. The resulting filtrate was analyzed using an Agilent 1290 Infinity UHPLC system (Agilent, Santa Clara, CA, USA) for chromatographic separation. Subsequent mass spectrometric analysis was performed on a 6500 QTRAP mass spectrometer (SCIEX, Framingham, MA, USA) operated in the positive ion mode. Quantitative analysis of target AAs was achieved using the multiple reaction monitoring (MRM) mode for specific ion pair detection (Table S2).

To analyze the medium- to long-chain fatty acid composition of meat, Tibetan sheep meat samples were thawed at 4 °C and mixed with dichloromethane-methanol solution (650463, Sigma, USA). After phase separation induced by water addition, the organic phase was collected and evaporated to dryness under a gentle nitrogen stream. The residue was reconstituted in n-hexane (650552, Sigma, St. Louis, MO, USA) spiked with methyl nonadecanoate (C19:0 methyl ester, Sigma-Aldrich, USA) as an internal standard, and subjected to methylation using 0.5 M methanolic sodium methoxide at 50 °C for 15 min. The reaction was then terminated by adding saturated ammonium chloride solution. Subsequently, distilled water was added, and the supernatant was collected, evaporated under nitrogen, and redissolved in n-hexane. The final extract was transferred into GC vials for gas chromatography-mass spectrometry (GC-MS) analysis. Separation was achieved on an Agilent 19091S-433UI capillary column (30 m × 250 μm × 0.25 μm) using gas chromatography coupled with an Agilent 5977B MSD system. Data acquisition was performed in both SCAN and selected ion monitoring (SIM) modes for accurate compound identification. Specifically, the SCAN mode (*m*/*z* 50–550) enabled acquisition of full mass spectra for preliminary identification by matching against the NIST 2020 mass spectral library with a similarity threshold >85%. Simultaneously, the SIM mode targeted characteristic fragment ions of specific fatty acid methyl esters (e.g., *m*/*z* 74, 87, 143 for saturated FAs; *m*/*z* 55, 67, 79, 81, 95, 108 for unsaturated FAs) to enhance detection sensitivity and provide confirmatory identification through retention time alignment and characteristic ion ratio consistency. Peak areas and retention times were processed using the MSD ChemStation software (version E.02.00.493, Agilent Technologies). Quantification of medium- and long-chain fatty acids was conducted based on an external standard curve.

### 2.6. Untargeted Metabolomics of Tibetan Sheep Meat

A 100 mg meat sample was undergone vacuum freeze-dried and ground for 90 s, then was mixed into 1.0 mL of extraction solution (Sigma-Aldrich, St. Louis, MO, USA). This mixture sat in the fridge overnight at 4 °C. The next day, it was vortexed a few times to make sure everything was well mixed, then spun down in a centrifuge at 10,000× *g* for 10 min. After that, the liquid on top was carefully filtered using a microporous membrane with 0.22 μm pores and transferred into a vial for Liquid Chromatography-Tandem Mass Spectrometry (LC-MS/MS) analysis. Chromatographic separation was performed using a Waters ACQUITY UPLC HSS T3 C18 column (2.1 × 100 mm, 1.8 µm). The chromatographic conditions were as follows: Mobile phase A was 0.1% formic acid aqueous solution, and mobile phase B was acetonitrile; gradient elution was applied (0–2 min: 5% B; 2–10 min: 5–95% B; 10–12 min: 95% B; 12–12.1 min: 95–5% B; 12.1–15 min: 5% B); flow rate was 0.3 mL/min, column temperature was 40 °C, and injection volume was 2 μL. Mass detection was conducted using an AB Sciex TripleTOF 6600 high-resolution mass spectrometer (AB Sciex LLC, Foster City, CA, USA) (instead of API 6500 QTRAP LC/MS/MS system (AB Sciex LLC, Foster City, CA, USA)) operating in electrospray ionization (ESI) source with simultaneous positive and negative ion modes. The mass spectrometry parameters were as follows: TOF-MS full-scan range was *m*/*z* 50–1200 (resolution ≥ 30,000 FWHM); data-dependent acquisition (DDA) mode was used to trigger MS/MS with a collision energy gradient of 20–50 eV; dynamic background subtraction technology was applied to reduce matrix interference.

Mass spectrometric data were acquired in both positive and negative ionization modes, with quality control (QC) samples systematically interspersed throughout the analytical sequence to ensure data reliability. The QC samples were prepared by pooling equal aliquots (10 μL) from each experimental sample to create a representative mixture. This pooled QC sample was processed identically to the individual samples, including all extraction and derivatization steps. During the analytical run, one QC sample was injected at the beginning of the sequence for system conditioning, followed by periodic insertion after every six experimental samples to monitor instrument stability and correct for signal drift. Orthogonal partial least squares-discriminant analysis (OPLS-DA) was performed to identify differentially abundant metabolites. The threshold for significant differences was set at variable importance in the projection (VIP) ≥ 1 and *p* < 0.05. The robustness and predictive ability of the OPLS-DA models were evaluated by the R2 and Q2 parameters. Furthermore, a permutation test (n = 200) was conducted to assess the risk of model overfitting. Differential metabolites were further analyzed using Kyoto Encyclopedia of Genes and Genomes (KEGG, https://academic.oup.com/nar/article/28/1/27/2384332 (accessed on 15 March 2025)) hypergeometric analysis, with pathways showing a Q-value ≤ 0.05 being considered significantly different.

### 2.7. Rumen Short-Chain Volatile Fatty Acids Analysis

The rumen fluid was carefully gathered into a 50 mL centrifuge tube, and its pH level was promptly assessed with a handheld pH meter (PH-STAR-11, Metris, Cologne, Germany). To measure the levels of SCFAs, a GC-MS system (Agilent, 7890B model), was utilized, featuring an Agilent DB-FFAP capillary column with dimensions of 30 μm × 250 μm × 0.25 μm.

### 2.8. Rumen Microbiota Analysis

Genomic DNA was extracted from rumen fluid samples using the HiPure Stool DNA Kit (Magen, Guangzhou, China). The integrity and concentration of the extracted DNA were assessed by agarose gel electrophoresis (DYY-6C, Beijing, China). PCR amplification (ETC811, Beijing, China) targeting the V3-V4 hypervariable region of the 16S rRNA gene was performed using the 341F (5′CCTACGGGNGGCWGCAG3′)-806R (5-GGACTACHVGGGTWTCTAAT-3′) primers. The amplification was conducted in a two-step PCR procedure. The resulting amplicons were purified with AMPure XP Beads and quantified using the StepOnePlus Real-Time PCR System (Applied Biosystems, Waltham, MA, USA). Sequencing was conducted using paired-end reads on the Illumina MiSeq PE250 platform (Illumina Inc., San Diego, CA, USA). To ensure data quality, raw reads were meticulously filtered through FASTP (Version 0.18.0) to yield clean tags. The clean tags were clustered into operational taxonomic units (OTUs) at a 97% similarity threshold using UPARSE (version 9.2.64). Finally, Krona (version 2.6) was employed to graphically represent the relative abundance of each taxonomic category. To identify shared and unique OTUs across experimental groups, Venn analysis was performed with the VennDiagram package (v1.6.16) in R. QIIME software (v1.9.1) was employed to assess alpha diversity, while beta diversity was evaluated through principal component analysis (PCA) using the Vegan package (v2.5.3) in R. Taxonomic annotations were performed against the SILVA database (version 138.1) to ensure accurate classification of rumen microbiota.

### 2.9. Statistical Analysis

All data were expressed as the mean ± standard error of the mean (SEM). Statistical analyses were performed using SPSS software (v26.0, IBM Corp., Armonk, NY, USA). One-way analysis of variance (ANOVA) was employed to assess the effects of dietary treatments on meat quality parameters, fatty acid profiles, and ruminal fermentation characteristics. When a significant overall effect was detected, Tukey’s honestly significant difference (HSD) post hoc test was used for pairwise comparisons among group means. A probability value of *p*-values < 0.05 was considered statistically significant.

To investigate the interrelationships between the rumen microbiota, muscle metabolome, and meat quality phenotypes, Spearman’s rank correlation analysis was conducted. Specifically, this analysis correlated the relative abundances of key bacterial genera, the levels of differential metabolites, and significant meat quality parameters. The resulting correlation matrix was visualized as a heatmap using the ‘pheatmap’ package in R (version 4.3.1).

## 3. Results

### 3.1. Meat Quality

As shown in Table 2, the LL muscle exhibited a significantly lower SF (*p* < 0.01) and hardness (*p* < 0.05) in the experimental groups than in the C group. Compared to the C group, the HMB and RES-HMB groups showed a significantly greater water-holding capacity (*p* < 0.01) and a noticeably higher a* value (*p* = 0.05). Conversely, the b* value in these two groups was considerably lower than that in the C group (*p* < 0.01). Meanwhile, the cooking loss was significantly lower in both the RES group and the RES-HMB group compared with the C group. Finally, adhesiveness and stickiness were significantly decreased (*p* < 0.05) in the experimental groups.

Proximate analysis revealed that the HMB and RES-HMB groups had significantly higher protein content compared to the C group (*p* < 0.01). At the same time, the fat content saw a significant drop across all the experimental groups (*p* = 0.05).

### 3.2. Genes Expression Related to Muscular Fiber Composition

**Figure 1:**
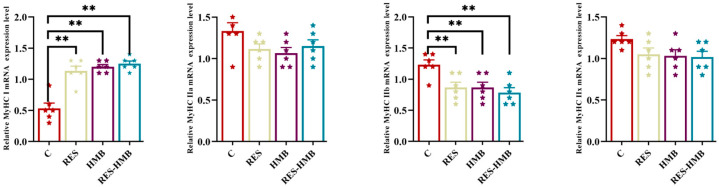
Impact of RES and/or HMB supplementation on *MyHC* gene expression in longissimus lumborum (LL) muscle of Tibetan Sheep. ** *p* < 0.01.

When it came to the LL muscle, the RES, HMB, and RES-HMB groups all showed a marked uptick in the expression of the myosin heavy chain I gene (*MyHC-I*) compared to the C group (*p* < 0.01). In contrast, *MyHC-IIb* expression took a nosedive (*p* < 0.01; Figure 1).

### 3.3. AA and Fatty Acid Composition of Meat

As summarized in Table 3, the concentrations of essential amino acids (EAAs) and non-essential amino acids (NEAAs) in the longissimus lumborum (LL) muscle were modulated by the dietary treatments. Within the EAAs, threonine content was significantly higher (*p* < 0.01) in all supplemented groups compared with the C group. Regarding NEAAs, glycine abundance exhibited a significant linear increase (*p* < 0.01) in response to the combined supplementation of RES and HMB. Moreover, the concentrations of serine and arginine were elevated (*p* < 0.05) in the RES and RES-HMB groups, while asparagine and histidine levels were higher (*p* < 0.05) in all treatment groups relative to the C group.

Dietary supplementation with RES and HMB, either individually or in combination, also significantly altered the fatty acid profile of the LL muscle (Table 4). Specifically, the contents of C20:3N6 in the RES and RES-HMB groups were significantly higher than those in the C group (*p* = 0.01), whereas the contents of C22:0 were significantly lower (*p* < 0.05). Furthermore, compared to the C group, the RES-HMB group showed a significant increase and decrease in the concentrations of C22:4N6 (*p* < 0.01) and C24:0 (*p* < 0.05), respectively.

### 3.4. Untargeted Metabolomic Analysis of Meat

**Figure 2:**
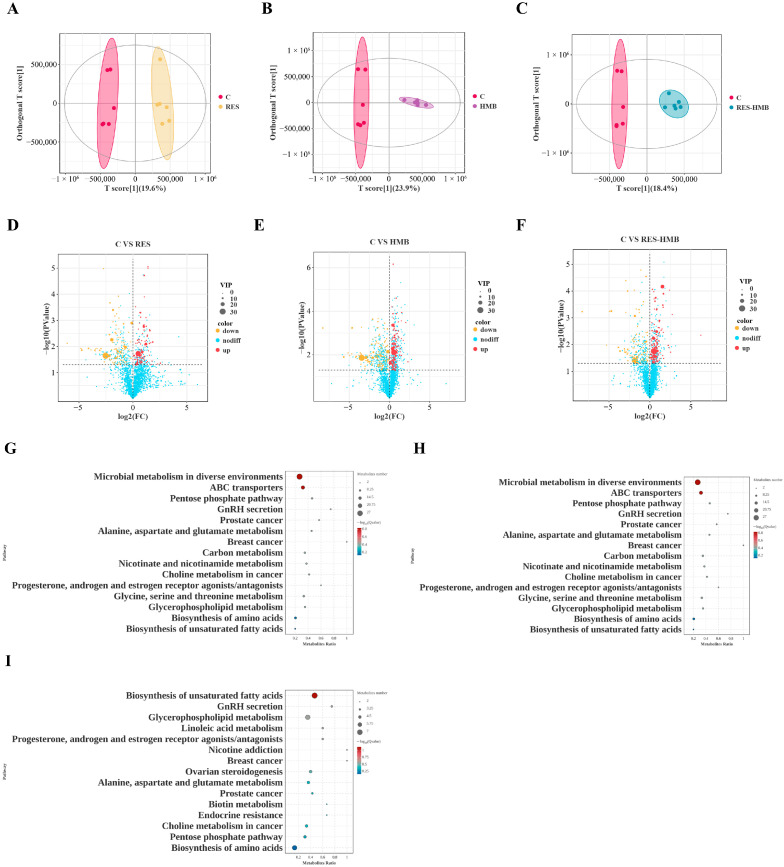
The Orthogonal partial least squares-discriminant analysis (OPLS-DA) score plots illustrate the distinctions between the C and RES groups (**A**), the C and HMB groups (**B**), and the C versus RES-HMB groups (**C**). Volcano plots were generated to highlight the differential metabolites identified in the C vs. RES (**D**), C vs. HMB (**E**), and C vs. RES-HMB (**F**) comparisons. Top 15 KEGG pathways enriched in comparisons: C vs. RES (**G**), C vs. HMB (**H**), C vs. RES-HMB (**I**).

Polar metabolites were examined using OPLS-DA, and cross-validation parameters between the C group and RES group (R2X = 0.396, R2Y = 0.979, Q2 = 0.450; Figure 2A and Figure S1A), the C group and HMB group (R2X = 0.515, R2Y = 0.999, Q2 = 0.783; Figure 2B and Figure S1B), and the C group and RES-HMB group (R2X = 0.516, R2Y = 0.997, Q2 = 0.824; Figure 2C and Figure S1C) were obtained. These findings demonstrated the clear separation between the C group and the experimental groups. The OPLS-DA model led to the identification of 714 differential metabolites (DMs). Specifically, compared to the C group, 200 DMs were identified in the RES group (up-regulated DMs = 91, down-regulated DMs = 109), 275 DMs in the HMB group (up-regulated DMs = 134, down-regulated DMs = 141), and 239 DMs in the RES-HMB group (up-regulated DMs = 127, down-regulated DMs = 112; Figure 2D–F).

The top 15 enriched pathways from each comparison (C group versus each experimental group) were selected for further analysis. Notably, four pathways were consistently enriched across all comparisons: Alanine, aspartate and glutamate metabolism; GnRH secretion; Biosynthesis of AAs; and Biosynthesis of unsaturated fatty acids (UFAs; Figure 2G–I). Moreover, 13 DMs related to these pathways were identified across all comparison groups: Beta-estradiol (M290T550_POS), Testosterone (M287T106_NEG), Argininosuccinic acid (M291T483_POS), D-aspartic acid (M115T459_NEG), L-aspartic acid (M115T384_NEG), N-acetyl-l-aspartic acid (M174T404_NEG), Arachidonic acid (M303T39_2_NEG), Cis-4,7,10,13,16,19-docosahexaenoic acid (M327T39_NEG), Linoleic acid (M279T39_NEG), Octadecanoic acid (M283T222_NEG), Chorismic acid (M191T409_POS), Ketoleucine (M129T57_NEG), and O-succinyl-l-homoserine (M200T182_NEG).

**Figure 3:**
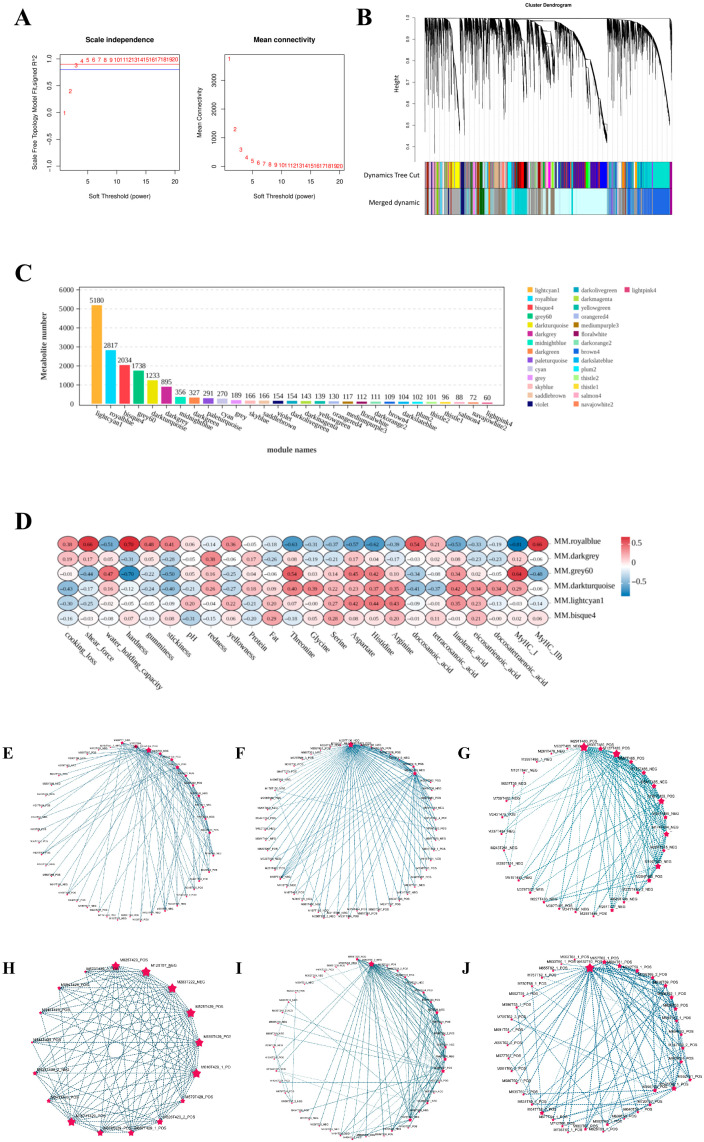
(**A**) The optimal soft-thresholding power was determined by analyzing its impact on mean connectivity. The red horizontal line indicates the signed R^2^ (Scale-free Topology Model Fit), and the blue horizontal line represents the threshold for the scale-free topology fit. (**B**) A hierarchical clustering dendrogram illustrates the grouping of differential metabolites (DMs). (**C**) A bar plot displays the distribution of DMs across identified modules. (**D**) A heatmap visualizes the correlations between module eigengenes and key phenotypic traits. (**E**–**J**) Correlation analysis between the top six modules with the highest number of metabolites and meat quality-related traits ((**E**) MM.light cyan, (**F**) MM.royal blue, (**G**) MM.bisque 4, (**H**) MM.gray 60, (**I**) MM.darkturquoise, (**J**) MM.dark gray).

Weighted gene co-expression network analysis (WGCNA) was employed to elucidate metabolite regulatory networks and identify pivotal metabolites associated with meat quality traits in Tibetan sheep. The scale-free topology model fit reached an optimum when the soft threshold power was set to 8, corresponding to a scale-free topology fit index of 0.90 (Figure 3A). A dendrogram with distinct colors representing different modules was generated to illustrate the clustering of metabolite modules (Figure 3B). Hierarchical clustering was performed to classify the metabolites into seven modules based on their expression patterns, and a module membership bar chart was generated to delineate the number of metabolites in each module (Figure 3C). The light cyan 1, royal blue, bisque 4, gray 60, dark turquoise, and dark gray modules contained the highest proportions of metabolites. In contrast, the bisque 4, cyan, and gray modules comprised the fewest. Correlation analysis demonstrated that the six largest modules were significantly correlated with key meat quality traits (Figure 3D). Subsequently, hub metabolites within these six key modules were identified using OmicShare tools (Version 3.0), defined as those possessing the top 100 intramodular connectivity rankings. Among these candidate metabolites, we identified 16 core metabolites, as follows: M115T384_NEG, M352T369_POS, and M747T176_POS (Figure 3E); M287T106_NEG and M513T400_NEG (Figure 3F); M291T483_POS, M191T409_POS, M127T485_POS, and M174T404_NEG (Figure 3G); M825T429_POS, M129T57_NEG, and M283T222_NEG (Figure 3H); and M303T39_2_NEG, M468T227_POS, and M279T39_NEG (Figure 3I), M632T60_POS (Figure 3J).

Based on the differential modules identified via WGCNA and the enriched pathways identified via KEGG analysis, Testosterone, Argininosuccinic acid, L-aspartic acid, N-acetyl-l-aspartic acid, Arachidonic acid, Linoleic acid, Octadecanoic acid, and Chorismic acid emerged as the key metabolites regulating meat traits in Tibetan sheep. The contents of Octadecanoic acid and Chorismic acid displayed a significant linear decrease in the experimental groups versus the C group, while the contents of other DMs displayed a significant linear increase.

### 3.5. Rumen Fermentation Parameters

**Figure 4:**
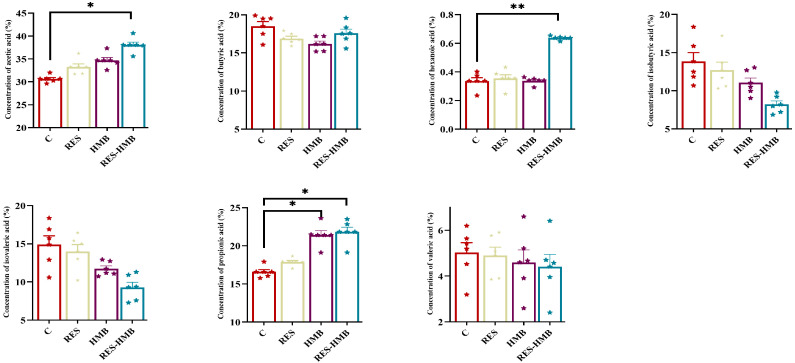
Impact of RES and HMB supplementation, both separately and together, on rumen short-chain fatty acids. * *p* < 0.05, ** *p* < 0.01.

Figure 4 revealed that the RES-HMB group exhibited a notably higher concentration of hexanoic acid (*p* < 0.01) and acetic acid (*p* < 0.05) when compared to the C group. Furthermore, both the HMB and RES-HMB groups demonstrated a significant rise in propionic acid levels (*p* < 0.05).

### 3.6. Ruminal Microbiota Composition

**Figure 5:**
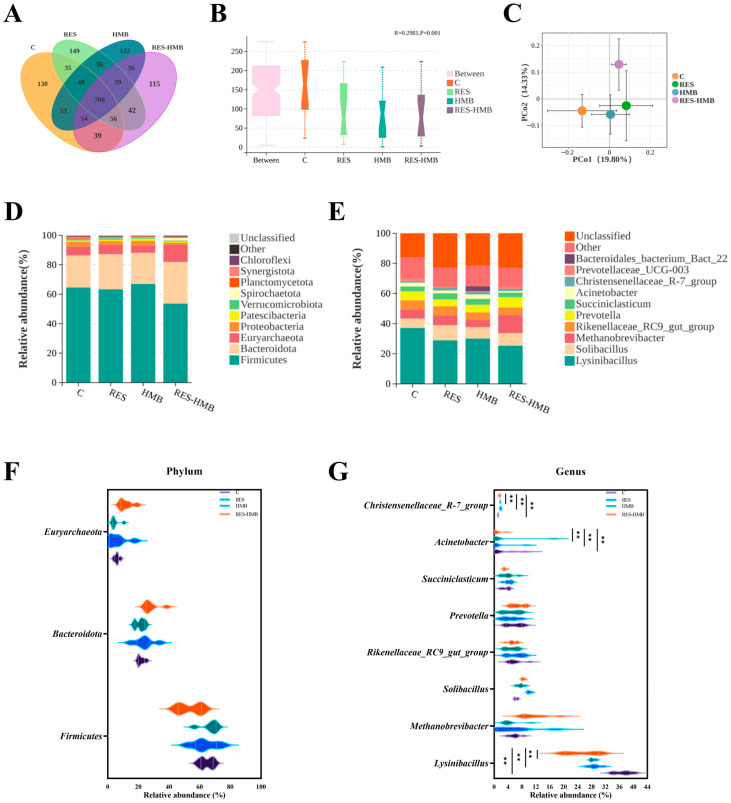
The Venn diagram depicting the operational taxonomic units (OTUs) of the ruminal microbiota among the four groups (**A**), followed by the analysis of variance (**B**) and Principal Coordinate Analysis (PCoA) plots representing the overall rumen microbiota samples (**C**). The relative abundance of bacterial communities is illustrated at both the phylum (**D**) and genus (**E**) levels across the four sample groups. The Comparison of microbial relative abundance at the phylum (**F**) and genus (**G**) levels as shown by the violin plot. ** *p* < 0.01.

Across the four groups of Tibetan sheep, rumen samples yielded 700 total OTUs, distributed as follows: C group (130 OTUs), RES group (149 OTUs), HMB group (122 OTUs), and RES-HMB group (115 OTUs; Figure 5A). ANOSIM (Figure 5B) and PCoA (Figure 5C) revealed a clear separation between the C group and the experimental groups.

At the phylum level, *Firmicutes*, *Bacteroidota* dominated the microbial populations in the rumen across all groups, followed by *Euryarchaeota* (Figure 5D,F). At the genus level, the abundance of *Solibacillus* and *Christensenellaceae_R-7_group* was significantly higher in all experimental groups than in the C group, while the abundance of *Lysinibacillus* was lower (Figure 5E,G).

### 3.7. Correlation Analysis

**Figure 6:**
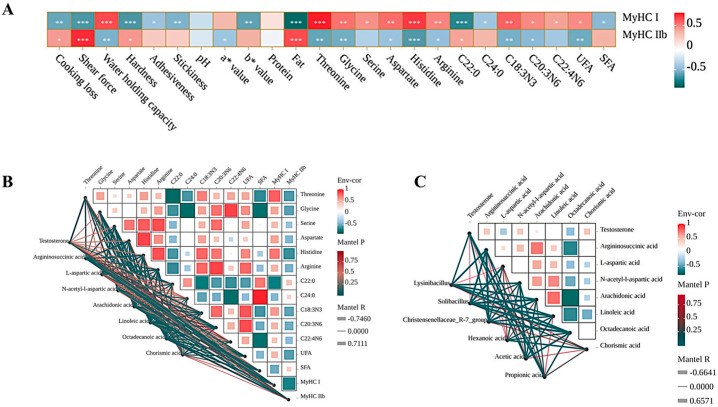
Spearman correlation analysis between gene expression and meat traits in the LL muscle of Tibetan sheep (**A**), meat phenotypes and rumen metabolites (**B**), ruminal short-chain fatty acids (SCFAs), microbiota composition, and core metabolites (**C**). * *p* < 0.05, ** *p* < 0.01, *** *p* < 0.001.

Spearman correlation analysis was conducted to understand the relationship between gene expression and meat traits in the LL muscle of Tibetan sheep (Figure 6A). The results showed that the expression of *MyHC-I* was positively correlated (*p* < 0.05) with meat quality parameters, the AAs, and UFA composition of the meat. However, the expression of *MyHC-II* had a negative correlation (*p* < 0.05) with these factors. Correlation analysis between meat traits and rumen metabolites revealed that the contents of Testosterone, Argininosuccinic acid, L-aspartic acid, N-acetyl-l-aspartic acid, Arachidonic acid, and Linoleic acid were positively correlated (*p* < 0.05) with the *MyHC-I* expression, edible and nutritional quality, AA contents, and polyunsaturated fatty acid (PUFA) contents of meat. Meanwhile, the contents of Octadecanoic acid and Chorismic acid showed a negative correlation (*p* < 0.05) with these parameters (Figure 6B). Furthermore, an integrated correlation analysis of ruminal SCFAs, microbiota, and core metabolites identified *Solibacillus*, *Christensenellaceae_R-7_group*, hexanoic acid, and acetic acid as key features showing positive associations. In contrast, the genus *Lysinibacillus* was negatively associated with the core metabolic profile (Figure 6C).

## 4. Discussion

As consumers’ priorities for meat products have shifted from quantity to quality, the development of high-quality meat through the dietary supplementation of green fodder additives during livestock and poultry rearing has become a growing area of research [35,36]. RES and HMB were selected for this study to investigate their potential mechanisms in modulating the meat quality of Tibetan sheep via rumen fermentation. RES undergoes partial microbial transformation in the rumen to bioactive metabolites including dihydroresveratrol, and 4-hydroxyphenylacetic acid, which can directly influence host metabolism [37,38]. In contrast, HMB appears to be primarily utilized as a metabolic precursor by rumen microorganisms, particularly enhancing AA synthesis pathways that support microbial protein production.

Meat quality is a complex concept, encompassing factors such as cooking loss, cooked meat yield, pH value, meat color, SF, and Water-Holding Capacity (WHC) [39]. Among these, color, particularly the a* and b* values, is a primary visual cue for consumers. The redness of meat increases when myoglobin is oxidized to produce oxygenated myoglobin. Type I muscle fibers are richer in mitochondria and myoglobin than Type II fibers [40]. Mitochondrial respiration competes with myoglobin for oxygen, but this competition decreases in later stages of meat storage, allowing normal myoglobin oxidation to resume and stabilizing the meat color [41]. The content of the *MyHC-I* isomer is positively correlated with muscle pH, whereas that of the *MyHC-II* isomer is negatively correlated with pH due to its higher glycolytic capacity and ATPase activity, which leads to the accumulation of more H^+^ [42]. Previous studies have shown that dietary supplementation with RES increases the content of Type I muscle fibers in beef cattle, and HMB supplementation in rats has shown similar effects [43,44]. Research has also indicated that these two feed additives can significantly improve the a* values and pH while reducing the b* value of meat [25,45]. These results agree with the present study, in which all experimental groups exhibited elevated pH levels relative to the C group. Furthermore, the HMB and RES-HMB groups displayed higher a* values and, conversely, diminished b* values. Evidence showed that both RES and HMB exhibit strong antioxidant properties, playing an important role in eliminating free radicals in muscle tissue and preventing lipid peroxidation, which in turn reducing cell membrane permeability and fluid loss [46,47,48]. Additionally, Type I muscle fibers contribute to improved WHC and reduced cooking loss [43]. Notably, some studies have found that meat texture parameters such as hardness, stickiness, and gumminess, which are directly related to mouthfeel, are negatively regulated by the WHC of meat [49,50]. Furthermore, oxidized fibers improve meat tenderness owing to their higher protein turnover and proteolytic activity, and their increased content may be attributed to the increased proportion of Type I fibers [51]. Furthermore, in ducks, dietary supplementation with RES has been shown to improve breast meat tenderness by reducing breast muscle diameter and cross-sectional area while increasing muscle density. Since lower SF values correspond to greater tenderness [16], these findings could explain why the WHC increased in the HMB and RES-HMB groups, cooking loss decreased in the RES and RES-HMB groups, and SF, hardness, stickiness, and gumminess were reduced across all experimental groups. Based on these findings, we speculate that supplementation with RES and HMB, either individually or jointly, can improve meat quality in Tibetan sheep by increasing the proportion of Type I muscle fibers.

The accretion of muscle mass and the enlargement of muscle fiber diameter are primarily driven by protein deposition, a process governed by the balance between protein synthesis and degradation, which is in turn modulated by nutritional induction [52]. Akt, a key protein, can phosphorylate TOR and promote protein synthesis by regulating essential proteins involved in mRNA translation [53]. It has been reported that RES can activate the PI3K/Akt pathway [54]. Interestingly, treating muscle cells with HMB also enhances Akt phosphorylation, which subsequently activates the mTORC1 signaling pathway to increase protein synthesis [55]. In addition, earlier research has shown that when RES is taken orally, it stimulates the formation of two intestinal metabolites: 4-hydroxyphenylacetic acid (4-HPA) and 3-hydroxyphenylpropionic acid (3-HPP). These metabolites have been found to enhance lipid metabolism in vitro and help mitigate obesity in mice that are given a high-fat diet [56]. Additionally, research has demonstrated that supplementation with HMB may improve metabolic capacity and fat utilization in muscle fibers, with fat being used as fuel to reduce protein breakdown [57]. Notably, muscle fibers contain multiple nuclei, which promote protein synthesis [58]. Fat accumulation is most prominent in the IIa and IIx/d fibers of the plantar muscles in obese mice [59]. In our study, compared to the C group, the concentration of protein was higher in the HMB and RES-HMB groups, while the concentration of fat tended to be lower in all experimental groups. Thus, we hypothesize that the observed increase in meat protein concentration may result from the concerted action of RES and HMB in activating the Akt pathway. This activation likely promotes protein synthesis while concurrently enhancing fat utilization as an energy substrate, potentially mediated by a shift in muscle fiber type composition. The precise mechanistic interplay warrants further investigation.

To validate the hypothesis that muscle fiber types correlate with the quality of meat, we selected four genes representing Type I (*MyHC-I*) and Type II (*MyHC-IIa*, *MyHC-IIx*, and *MyHC-IIb*) muscle fibers for quantitative analysis. The results revealed that, compared to the C group, *MyHC-I* expression was significantly up-regulated in the experimental groups, while *MyHC-IIb* expression was down-regulated. The activation of the PI3K/Akt pathway by both RES and HMB represents a direct molecular mechanism promoting slow-twitch fiber development [60]. Concurrently, microbiome-generated metabolites likely contribute indirectly through systemic signaling and epigenetic modifications. The correlation between specific microbial taxa (particularly *Christensenellaceae_R-7_group*) and *MyHC I* expression supports this microbiome-mediated influence.

As precursors for the flavor of cooked meat, AAs such as threonine, glycine, serine, and asparagine contribute to the sweetness, umami, bitterness, and other flavors found in animal meat products [61]. Threonine and serine are often used for protein synthesis in muscles and for increasing backfat thickness due to their low cost [62]. Glycine can enhance the a* value of pork by promoting the synthesis of heme, an important component of myoglobin [63]. Moreover, glycine enhances pork tenderness via conversion of Type II to Type I muscle fibers [64]. Meanwhile, arginine plays a key role in activating the mTOR signaling pathway in mice to up-regulate the slow fiber gene (*MyHC-I*) in skeletal muscle [65]. It also activates the protein kinase B (Akt)/mTOR signaling pathway to trigger muscle protein synthesis [66]. Additionally, dietary supplementation with arginine reduces the SF of muscle tissues by weakening the myofibril structure [67], while damaging fine filaments and/or actin bonds to increase meat tenderness [68]. Additionally, it decreases cooking loss by enhancing myosin solubility [69]. L-histidine is involved in the synthesis of neuronal histamine, which helps reduce the b* value of broiler meat at 45 min postmortem [70]. Dietary D-aspartate supplementation can increase the number of mitochondria in muscle fibers, maintaining the redness of meat and preventing glycogen storage, and thus lowering pH by increasing the production of lactic acid [71,72]. Previous research has reported that RES and HMB improve the content of AAs, which is consistent with our findings [16,73,74,75]. This is further supported by our non-targeted metabolomics data, which revealed a significant up-regulation of the “Biosynthesis of AAs” pathway in the experimental groups. Moreover, core metabolites associated with this pathway, along with *MyHC-I* gene expression, showed a positive correlation with AA content. Collectively, these results suggest that RES and HMB, individually or in combination, may enhance the AA content in meat and/or activate endogenous AA biosynthesis. This metabolic shift potentially promotes a higher proportion of Type I muscle fibers, ultimately improving meat palatability and nutritional quality.

Fatty acids serve as fundamental building blocks for lipids, not only forming triglycerides upon esterification with glycerol but also constituting critical components of cellular membranes and serving as key energy reservoirs [76,77]. It is important to note that UFAs are positively correlated with several meat quality traits, including meat color, tenderness, marbling, and flavor, while saturated fatty acids (SFAs) are less favorable for meat quality [78,79,80]. The excessive intake of SFAs can lead to atherosclerosis and harm overall health [81], while replacing saturated fats with PUFAs appears to be an effective strategy for reducing the risk of cardiovascular disease [82]. Dietary nutrient intake can impact the fatty acid composition of animal products [83]. One study showed that feeds containing antioxidants, such as polyphenols, help maintain the content of UFAs in muscle tissue [84]. Another study indicated that the beneficial effects of RES and PUFAs may overlap, with RES playing a key role in promoting PUFA synthesis [85]. Additionally, research in broilers concluded that diets containing 0.05% HMB may reduce the content of SFAs, thereby decreasing meat firmness [75]. Furthermore, PUFAs such as linoleic acid, α-linolenic acid, γ-linolenic acid, arachidonic acid, docosahexaenoic acid, and eicosapentaenoic acid can activate PPARα [86], which can significantly increase the number of Type I muscle fibers through the muscle-specific over expression of PPARδ [87]. These findings are in line with our experimental findings, which showed a significant increase in the contents of PUFAs, such as linolenic acid (C18:3N3), eicosatrienoic acid (C20:3N6), and docosatetraenoic acid (C22:4N6), in the experimental groups compared to the C group. Notably, non-targeted metabolomics revealed that the KEGG pathway for “Biosynthesis of UFAs” was significantly up-regulated in all experimental groups. Moreover, the core metabolites in this pathway, the expression of *MyHC-I*, and the levels of PUFAs showed a positive relationship with each other. Collectively, these findings support the inference that dietary supplementation with RES and HMB, either individually or in combination, enhances meat quality in Tibetan sheep by activating the biosynthetic pathway for UFAs. This up-regulation increases intramuscular UFA deposition, which in turn promotes the development of Type I muscle fibers.

Microorganisms involved in rumen fermentation play a critical role in host metabolism, exchanging nutrients and information with the host [88]. In the present study, *Firmicutes*, *Bacteroidota*, and *Euryarchaeota* were identified as the dominant bacterial phyla across all experimental groups, consistent with previous findings [89]. At the genus level, the abundance of *Christensenellaceae_R-7_group* and *Solibacillus* was significantly higher in all experimental groups than in the C group. *Christensenellaceae_R-7_group* utilizes cellulase and hemicellulase to degrade cellulose and convert it into SCFAs [90]. Furthermore, as a member of the *Firmicutes* phylum, *Christensenellaceae_R-7_group* is also involved in regulating immune cell numbers and participating in metabolism, including AA metabolism [91,92,93]. *Solibacillus*, known for its surfactant properties, facilitates biofilm formation [94]. Deng et al. found that *Solibacillus* abundance is closely linked to various metabolites, including SCFAs and Aas [95]. SCFAs, such as acetic acid, propionic acid, and hexanoic acid are derived via the fermentation of dietary fiber by a range of bacteria. These SCFAs exert direct effects on meat quality: propionic acid supports muscle energy metabolism to stabilize pH; butyric acid reduces intestinal inflammation to enhance AA absorption; acetic acid serves as a precursor for intramuscular fatty acid synthesis. These SCFAs regulate lipid metabolism and can improve carcass traits and meat quality [96]. In the rumen, microorganisms break down feed, kicking off the production of propionic and acetic acids. These acids then act as the building blocks for branched-chain fatty acids (BCFAs). Typically saturated, BCFAs come in two main flavors: those with a single branch and those sporting multiple branches [97]. Notably, *Prevotella* abundance decreases in RES/HMB alone groups but increases in RES-HMB group—HMB provides methyl donors to restore *Prevotella* proliferation, enhancing amino acid release and explaining the highest AA content in this group; *Methanobrevibacter* abundance is highest in RES-HMB group, but HMB competes for hydrogen substrates to reduce methane production, maintaining rumen redox balance for stable SCFAs synthesis. Previous studies have shown that RES, as a compound with low bioavailability, can generate SCFAs through the gut microbiota, and HMB also has similar functions [98,99]. Our results are consistent with these reports, demonstrating that dietary supplementation with RES and HMB, either individually or in combination, enhanced ruminal SCFA levels in Tibetan sheep. Furthermore, our study revealed that these probiotics and SCFAs play a beneficial role in regulating the metabolites essential for the synthesis of AAs and UFAs. Collectively, these findings suggest that ruminal beneficial bacteria may ferment RES and HMB to produce SCFAs, which in turn activate metabolic pathways related to AA and UFA biosynthesis, ultimately contributing to the improvement of meat quality in Tibetan sheep.

## 5. Conclusions

In summary, this study demonstrates that RES and HMB supplementation, either alone or in combination, improves the edible and nutritional quality of Tibetan sheep meat by significantly up-regulating *MyHC I* expression. This enhancement is primarily mediated through two interconnected mechanisms: first, the direct activation of metabolic pathways related to AAs and PUFAs, such as alanine, aspartate, glutamate metabolism, biosynthesis of amino acids, and biosynthesis of unsaturated fatty acids; second, the enrichment of beneficial rumen microbiota (*Christensenellaceae_R-7_group* and *Solibacillus*), whose fermentation-derived SCFAs further facilitate the accumulation of AAs and PUFAs. These metabolites are pivotal for promoting *MyHC I* expression. Our findings offer novel strategies for quality improvement in Tibetan sheep and support the use of green feed additives in sustainable animal production.

## Figures and Tables

**Table 1:** Dietary concentrate composition and nutrient levels (dry matter basis).

	Items	Content (%)
Ingredient	Corn	51.50
	Soybean meal	2.00
	Rapeseed meal	12.80
	Cottonseed meal	2.00
	Palm meal	25.00
	NaCl	1.00
	Limestone	1.00
	Baking soda	0.10
	Premix ^1^	4.60
	Total	100.00
Nutrient levels	Digestible energy (MJ/kg) ^2^	12.71
	Crude protein	14.27
	Ether extract	3.29
	Neutral detergent fiber	26.70
	Acid detergent fiber	19.97
	Ca	0.86
	P	0.40

^1^ The premix provides 18 mg of Cu, 66 mg of Fe, 30 mg of Zn, 48 mg of Mn, 0.36 mg of Se, 0.6 mg of I, 0.24 mg of Co, 24,000 IU of VA, 4800 IU of VD, and 48 IU of VE per kilogram of feed. ^2^ Digestive energy is calculated, while the rest are measured values.

**Table 2:** Effect of adding resveratrol and β-hydroxy-β-methylbutyric acid on the Edible and nutritional quality of meat of Tibetan sheep.

Items	Group				*p*-Value
	C	RES	HMB	RES-HMB	
Eye muscle area	24.00 ± 1.10	25.60 ± 1.36	22.87 ± 1.55	25.40 ± 1.28	0.445
pH	6.46 ± 0.07	6.51 ± 0.11	6.43 ± 0.07	6.59 ± 0.05	0.520
L*	32.48 ± 1.51	35.19 ± 1.82	34.09 ± 0.60	33.32 ± 2.83	0.769
a*	21.72 ± 1.73 ^b^	22.28 ± 1.38 ^b^	26.89 ± 1.62 ^a^	27.00 ± 1.75 ^a^	0.050
b*	18.75 ± 0.53 ^a^	17.06 ± 0.73 ^ab^	14.77 ± 1.19 ^b^	10.18 ± 0.63 ^c^	<0.001
Thawing loss rate (%)	1.99 ± 0.32	1.63 ± 0.32	1.81 ± 0.45	1.47 ± 0.30	0.751
Cooking loss rate (%)	54.98 ± 1.66 ^a^	35.84 ± 1.32 ^bc^	53.09 ± 1.06 ^a^	33.95 ± 0.92 ^b^	<0.001
Shear force (N)	56.74 ± 3.10 ^a^	41.64 ± 1.72 ^b^	43.52 ± 2.01 ^b^	38.71 ± 1.76 ^b^	0.001
Water holding capacity (N)	10.95 ± 0.64 ^b^	15.08 ± 0.91 ^b^	20.54 ± 1.39 ^a^	23.09 ± 1.92 ^a^	<0.001
Hardness (N)	22.46 ± 1.20 ^a^	19.97 ± 0.39 ^b^	14.66 ± 0.34 ^c^	18.36 ± 0.68 ^b^	<0.001
Stickness (mj)	0.77 ± 0.05 ^a^	0.55 ± 0.04 ^b^	0.55 ± 0.08 ^b^	0.53 ± 0.03 ^b^	0.015
Cohesion (mj)	0.39 ± 0.03	0.44 ± 0.03	0.49 ± 0.06	0.48 ± 0.03	0.277
Elasticity (mm)	3.15 ± 0.06	3.38 ± 0.15	3.21 ± 0.13	3.49 ± 0.10	0.174
Chewiness (mj)	42.30 ± 0.98	37.93 ± 1.57	38.27 ± 0.82	37.20 ± 2.63	0.160
Adhesiveness (N)	11.19 ± 0.90 ^a^	9.03 ± 0.68 ^ab^	8.48 ± 0.95 ^b^	7.61 ± 0.68 ^b^	0.034
Protein (%)	20.93 ± 0.17 ^b^	20.33 ± 0.38 ^b^	21.07 ± 0.21 ^a^	21.87 ± 0.31 ^a^	0.008
Moisture (%)	74.10 ± 0.15	75.67 ± 0.68	74.13 ± 0.45	74.53 ± 0.12	0.052
Fat (%)	2.23 ± 0.01 ^a^	1.20 ± 0.03 ^b^	1.20 ± 0.00 ^b^	1.13 ± 0.03 ^c^	<0.001
Ash (%)	0.80 ± 0.03	0.63 ± 0.11	0.73 ± 0.04	0.77 ± 0.06	0.340

Note: In the same row, values with no letter or the same letter superscripts mean no significant difference (*p* > 0.05), while with different small letter superscripts mean significant difference (*p* < 0.05).

**Table 3:** Effect of addition of resveratrol and β-hydroxy-β-methylbutyric acid on amino acids in the longissimus lumborum (LL) muscle of Tibetan sheep.

Items	Group				*p*-Value
	C	RES	HMB	RES-HMB	
EAA					
Lysine (µmol/kg)	19.70 ± 2.74	34.20 ± 6.19	20.81 ± 0.52	23.73 ± 2.42	0.076
Methionine (µmol/kg)	9.67 ± 2.74	14.43 ± 2.97	10.07 ± 0.95	7.55 ± 1.20	0.227
Leucine (µmol/kg)	24.20 ± 3.94	32.18 ± 3.34	25.09 ± 0.96	22.70 ± 2.07	0.164
Isoleucine (µmol/kg)	17.29 ± 3.66	23.81 ± 1.73	18.55 ± 1.27	19.78 ± 1.73	0.282
Valine (µmol/kg)	20.34 ± 2.02	28.13 ± 3.58	19.92 ± 0.55	23.92 ± 1.81	0.105
Threonine (µmol/kg)	30.92 ± 1.44 ^c^	46.72 ± 2.17 ^b^	56.29 ± 1.65 ^a^	60.90 ± 4.58 ^a^	0.001
Asparagine (µmol/kg)	0.49 ± 0.03	0.47 ± 0.09	0.39 ± 0.02	0.66 ± 0.14	0.221
Glutamine (µmol/kg)	0.62 ± 0.05	0.67 ± 0.12	0.62 ± 0.02	0.53 ± 0.08	0.644
Cysteine (µmol/kg)	0.03 ± 0.01	0.04 ± 0.02	0.03 ± 0.00	0.03 ± 0.01	0.865
Tryptophan (µmol/kg)	0.20 ± 0.04	0.29 ± 0.06	0.23 ± 0.01	0.14 ± 0.01	0.137
NEAA					
Glutamate (µmol/kg)	6.21 ± 1.50	4.08 ± 0.96	4.51 ± 0.54	6.34 ± 0.71	0.488
Glycine (µmol/kg)	93.33 ± 1.70 ^b^	126.09 ± 10.47 ^b^	103.31 ± 4.78 ^b^	181.84 ± 11.10 ^a^	0.003
Aspartate (µmol/kg)	8.20 ± 1.11 ^b^	36.17 ± 0.45 ^a^	24.45 ± 1.22 ^a^	16.92 ± 0.46 ^a^	0.001
Arginine (µmol/kg)	16.10 ± 1.27 ^b^	27.87 ± 1.36 ^a^	17.75 ± 0.84 ^b^	25.06 ± 1.56 ^a^	0.001
Serine (µmol/kg)	31.66 ± 3.71 ^b^	54.38 ± 7.95 ^a^	36.16 ± 2.62 ^b^	42.19 ± 1.61 ^a^	0.040
Tyrosine (µmol/kg)	20.12 ± 3.22	30.21 ± 6.87	18.47 ± 1.32	20.24 ± 2.60	0.236
Histidine (µmol/kg)	79.09 ± 1.38 ^b^	131.00 ± 4.86 ^a^	107.91 ± 3.06 ^a^	113.16 ± 3.29 ^a^	0.001
Proline (µmol/kg)	13.76 ± 0.85	15.81 ± 1.72	13.20 ± 0.11	16.02 ± 1.05	0.243
Sarcosine (µmol/kg)	33.63 ± 0.30	35.97 ± 6.02	31.39 ± 0.49	30.91 ± 0.14	0.639
Cystine (µmol/kg)	14.43 ± 1.42	19.89 ± 2.22	15.71 ± 1.39	17.15 ± 2.20	0.264

Note: In the same row, values with no letter or the same letter superscripts mean no significant difference (*p* > 0.05), while with different small letter superscripts mean significant difference (*p* < 0.05).

**Table 4:** Effect of RES and HMB on medium to long chain fatty acids in the longissimus lumborum (LL) muscle of Tibetan sheep.

Items	Group				*p*-Value
	C	RES	HMB	RES-HMB	
C8:0 (µg/g)	1.72 ± 0.11	1.56 ± 0.26	1.87 ± 0.22	1.36 ± 0.18	0.354
C11:0 (µg/g)	0.51 ± 0.36	0.23 ± 0.13	0.23 ± 0.04	0.53 ± 0.04	0.563
C21:0 (µg/g)	6.64 ± 0.70	8.10 ± 1.00	6.97 ± 0.92	7.50 ± 0.76	0.655
C22:0 (µg/g)	287.61 ± 6.26 ^a^	261.59 ± 3.18 ^b^	265.02 ± 4.29 ^ab^	246.61 ± 12.40 ^bc^	0.028
C23:0 (µg/g)	198.47 ± 3.70	195.23 ± 13.43	192.39 ± 14.45	173.67 ± 8.09	0.419
C24:0 (µg/g)	772.17 ± 14.43 ^a^	761.16 ± 23.38 ^a^	750.19 ± 39.70 ^a^	628.12 ± 26.89 ^b^	0.020
C15:1N5 (µg/g)	267.17 ± 2.15	244.15 ± 11.22	252.72 ± 17.77	237.01 ± 7.92	0.333
C18:3N3 (µg/g)	113.99 ± 9.70 ^b^	165.81 ± 19.17 ^a^	143.33 ± 7.03 ^a^	168.91 ± 2.67 ^a^	0.031
C20:3N6 (µg/g)	70.61 ± 4.91 ^b^	89.57 ± 2.12 ^a^	72.06 ± 4.27 ^b^	91.02 ± 4.14 ^a^	0.010
C22:5N3 (µg/g)	8.50 ± 0.29	8.26 ± 0.60	8.72 ± 1.13	9.16 ± 0.66	0.843
C22:4N6 (µg/g)	11.75 ± 0.40 ^b^	12.22 ± 0.61 ^b^	12.03 ± 1.04 ^b^	22.47 ± 2.12 ^a^	0.001
C22:5N6 (µg/g)	42.58 ± 3.85	41.36 ± 0.61	42.58 ± 1.41	43.93 ± 3.04	0.916
SFA	1267.11 ± 9.64 ^a^	1227.86 ± 16.85 ^a^	1216.67 ± 26.11 ^b^	1057.79 ± 10.34 ^b^	<0.001
UFA	514.60 ± 8.40 ^b^	561.36 ± 2.09 ^a^	531.19 ± 12.88 ^b^	572.50 ± 2.10 ^a^	<0.001

Note: In the same row, values with no letter or the same letter superscripts mean no significant difference (*p* > 0.05), while with different small letter superscripts mean significant difference (*p* < 0.05).

## Data Availability

The data reported in this paper have been deposited in the OMIX, (https://ngdc.cncb.ac.cn/omix (accessed on 1 June 2025), accession: OMIX009140) and NCBI SRA (https://www.ncbi.nlm.nih.gov/sra/ (accessed on 1 June 2025), accession: PRJNA1108485).

## Supplementary Materials

The following supporting information can be downloaded at: https://www.mdpi.com/article/10.3390/microorganisms13122845/s1, Table S1: Primers used in qRT-PCR; Table S2: MRM Detection Parameters of Target Amino Acids; Figure S1: Permutation tests were conducted to validate the PLS-DA models for the same group comparisons.

## Abbreviations

The following abbreviations are used in this manuscript:

BCFAs	Branched-chain fatty acids
DMs	Differential metabolites
EAAs	Essential AAs
GC-MS	Gas chromatography-mass spectrometry
HMB	β-hydroxy-β-methylbutyric acid
KEGG	Kyoto Encyclopedia of Genes and Genomes
LC-MS/MS	Liquid Chromatography-Tandem Mass Spectrometry
LIT	Linear ion trap
LL	Longissimus lumborum
Met	Methionine
NEAAs	Non-essential AAs
OPLS-DA	Orthogonal partial least squares-discriminant analysis
OTUs	Operational taxonomic units
PCA	Principal component analysis
PUFA	Polyunsaturated fatty acid
QC	quality control
QQQ	Triple quadrupole
RES	Resveratrol
SCFAs	Short-chain fatty acids
SEM	Standard error of the mean
SF	Shear force
SFAs	Saturated fatty acids
UFA	Unsaturated fatty acid
VIP	Variable importance in the projection
WHC	Water-Holding Capacity
WGCNA	Weighted gene co-expression network analysis
3-HPP	3-hydroxyphenylpropionic acid
4-HPA	4-hydroxyphenylacetic acid
