# Supplementary material for: Metabolome Combined with 16S rDNA Sequencing Reveals a Novel Mechanistic Insight into the Collaboration of Resveratrol and β-Hydroxy-β-Methylbutyric Acid in Regulating the Meat Quality of Tibetan Sheep Through Altering Rumen Microbiota
Source: Microorganisms. 2025 Dec 15;13(12):2845. doi: 10.3390/microorganisms13122845 (PMC12735672; doi:10.3390/microorganisms13122845)
Supplement: Supplementary file 1 [file microorganisms-13-02845-s001.zip › microorganisms-3956407-supplementary.pdf]

Table S1. Primers used in qRT-PCR

Gene	Primer sequence (5′ – 3′)	Product size (bp)
MyHC I	F:ATCGCTGAATCCCAGGTCAA<br>R:ACCAAGATGTGGCACGGCTA	92
MyHC IIa	F:CACCCTGGAGCAGACAGAGA<br>R:TCCCTGGATTTGCGTGATG	148
MyHC IIx	F:AGGGCATCGCTGGAACAGAC<br>R:CAGAAGCTGCACACGCTCAC	84
MyHC IIb	F:TTTCCAGACCGTGTCTGCTC<br>R:GGGATGATGCAGCGTACAAAG	96
GAPDH	F:GATGGTGAAGGTCGGAGTGAAC<br>R:GTCATTGATGGCGACGATGT	100

Table S2. MRM Detection Parameters of Target Amino Acids.

Amino Acid	Precursor Ion (m/z)	Quantifier Ion (m/z)	Collision Energy (eV)	Quantifier Ion (m/z)	Collision Energy (eV)
Lysine	366.2	366.2→198.1	22	366.2→129.0	28
Glutamic Acid	337.1	337.1→179.0	18	337.1→141.0	24
Methionine	358.2	358.2→189.1	20	358.2→151.0	26

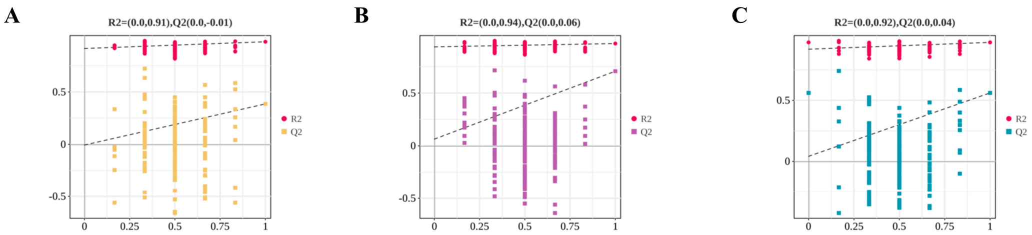

Figure S1. permutation tests were conducted to validate the PLS-DA models for the same group comparisons: C vs RES (A), C vs HMB (B), and C vs RES-HMB (C).
